# Network-Based Integration of Disparate Omic Data To Identify "Silent Players" in Cancer

**DOI:** 10.1371/journal.pcbi.1004595

**Published:** 2015-12-18

**Authors:** Matthew Ruffalo, Mehmet Koyutürk, Roded Sharan

**Affiliations:** 1 Department of Electrical Engineering and Computer Science, Case Western Reserve University, Cleveland, Ohio, United States of America; 2 Center for Proteomics and Bioinformatics, Case Western Reserve University, Cleveland, Ohio, United States of America; 3 School of Computer Science, Tel Aviv University, Tel Aviv, Israel; University of Southern California, UNITED STATES

## Abstract

Development of high-throughput monitoring technologies enables interrogation of cancer samples at various levels of cellular activity. Capitalizing on these developments, various public efforts such as The Cancer Genome Atlas (TCGA) generate disparate omic data for large patient cohorts. As demonstrated by recent studies, these heterogeneous data sources provide the opportunity to gain insights into the molecular changes that drive cancer pathogenesis and progression. However, these insights are limited by the vast search space and as a result low statistical power to make new discoveries. In this paper, we propose methods for integrating disparate omic data using molecular interaction networks, with a view to gaining mechanistic insights into the relationship between molecular changes at different levels of cellular activity. Namely, we hypothesize that genes that play a role in cancer development and progression may be implicated by neither frequent mutation nor differential expression, and that network-based integration of mutation and differential expression data can reveal these “silent players”. For this purpose, we utilize network-propagation algorithms to simulate the information flow in the cell at a sample-specific resolution. We then use the propagated mutation and expression signals to identify genes that are not necessarily mutated or differentially expressed genes, but have an essential role in tumor development and patient outcome. We test the proposed method on breast cancer and glioblastoma multiforme data obtained from TCGA. Our results show that the proposed method can identify important proteins that are not readily revealed by molecular data, providing insights beyond what can be gleaned by analyzing different types of molecular data in isolation.

## Author Summary

Identification of cancer-related genes is an important task, made more difficult by heterogeneity between samples and even within individual patients. Methods for identifying disease-related genes typically focus on individual data sets such as mutational and differential expression data, and therefore are limited to genes that are implicated by each data set in isolation. In this work we propose a method that uses protein interaction network information to integrate mutational and differential expression data on a sample-specific level, and combine this information across samples in ways that respect the commonalities and differences between distinct mutation and differential expression profiles. We use this information to identify genes that are associated with cancer but not readily identifiable by mutations or differential expression alone. Our method highlights the features that significantly predict a gene’s association with cancer, shows improved predictive power in recovering cancer-related genes in known pathways, and identifies genes that are neither frequently mutated nor differentially expressed but show significant association with survival.

## Introduction

The sequencing revolution of the last decade is producing vast amounts of data with clinical relevance. However, translating these data to biomedical understanding remains a formidable challenge due to the typically low statistical power associated with sequencing studies, disease heterogeneity, experimental limitations and more. A promising strategy to circumvent some of these problems is the integration of sequence data with other types of “omic” data [1]. In the context of cancer, comprehensive data generation efforts such as The Cancer Genome Atlas (TCGA) and and the COSMIC cancer gene census [2] provide excellent opportunities in this regard, since they interrogate large sets of samples for multiple types of omic data.

An important and well-studied problem in this field is the prioritization of genes for specific diseases. State-of-the-art methods for tackling this problem rely on the observation that proteins causing similar diseases tend to lie close to one another in a protein-protein interaction network. We have previously devised prioritization methods that start from known causal proteins and propagate their signal in the network to predict novel causal proteins [3, 4]. Here, we aim to harness the network propagation methodology to the integration of multiple omic data types in the context of cancer, with a view to gaining mechanistic insights into the relationship between molecular changes at different levels of cellular activity.

### Related Work

In recent years, there have been substantial efforts in integrating multiple omic data types that provide information on cancer pathogenesis and progression, with a view to predicting patient outcome, identifying drug targets, and understanding the functional relationships among key players in cancer. In the context of predicting patient outcome, Hofree *et al*. [5] used a network propagation based strategy to incorporate the functional relationships among mutated genes into the clustering of patients. They showed that the resulting clustering correlates with patient outcomes better than the clustering of patients according to mutation data alone. Similarly, several groups demonstrated that integration of transcriptomic data with protein-protein interaction networks leads to the identification of protein subnetworks that serve as reliable markers for the prediction of survival in such cancers as glioblastoma multiforme [6] and ovarian cancer [7].

In the context of understanding the functional relationships among key players in cancer, enrichment-based approaches aimed at identifying significantly mutated pathways provide insights into how different mutations influences similar biological processes [8]. Analysis of mutually exclusive mutations further elucidate the functional relationships among mutated genes by interpreting mutual exclusivity among mutations in the context of networks, thereby recovering key functional modules that provide systems-level insights into the mechanisms of pathogenesis [9]. Integration of sequence data with gene expression data based on eQTL analysis is also shown to be effective in the identification of cancer-related pathways [10]. These studies establish that the addition of network information can enhance predictive power in many applications, but most of these methods focus on a single data type in addition to network relationships. Though previous studies combine mutational or differential expression data with protein interaction networks, few use network information to integrate mutational *and* expression data. In particular, Nibbe *et al*. [11] propose a method that integrates protein expression data with mRNA expression data, with the purpose of extending the scale of of proteomic data that has limited coverage of the proteome. In Nibbe *et al*.’s study proteomic and transcriptomic data from different patients is used to integrate mRNA-level gene expression and protein expression data. However, efforts like TCGA make it possible to extract multiple types of omic data (mutation, mRNA expression, microRNA expression etc.). In this study, we aim to develop an algorithmic framework for the integration of these multi-omic data at the level of individual samples.

### Driving Hypothesis and Computational Workflow

We stipulate that during pathogenesis of cancer, mutations in up-stream proteins may lead to transcriptional dysregulation of down-stream genes. Similarly, transcriptional dysregulation of some processes may lead to conservation of certain mutations during neoplastic evolution. The dynamics of the interplay between genomic mutations and transcriptional dysregulation likely involves signaling proteins (e.g., kinases, phosphatases, transcription factors) that mediate the relationship between mutated genes and dysregulated gene products. However, due to limitations in proteomic and phosphoproteomic screening [12], the changes in those mediator proteins may not be readily detectable from genomic and transcriptomic data alone. We propose that such “silent” proteins can be detected by integrating mutation and differential expression data in a network context, since these proteins are likely to be in close proximity to both mutated and differentially expressed proteins in the network of protein-protein interactions (PPIs).

**Fig 1:**
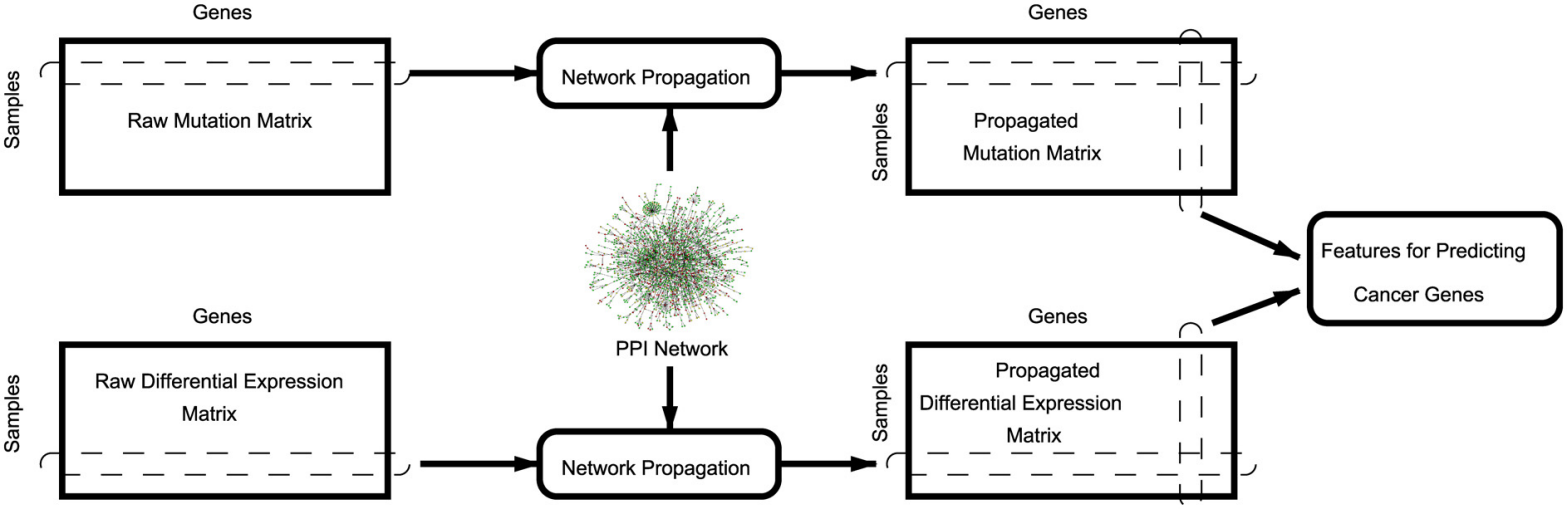
The workflow of the proposed algorithmic pipeline that integrates mutation, gene expression, and protein-protein interaction (PPI) data to test the driving hypothesis and identify causal genes.

Based on our hypothesis, we develop an algorithmic workflow aimed at quantifying the proximity of all proteins in the human proteome to the products of mutated and differentially expressed genes in each sample. The proposed workflow is illustrated in Fig 1. Here, our emphasis is on utilizing sample-specificity to be able to deal with molecular heterogeneity of pathogenesis at the population level. In order to utilize sample-specific data, we use network propagation to separately score proteins based on their network proximity to 1) mutated and 2) differentially expressed genes in each sample. This procedure provides us with two vectors in the space of samples for each protein: a “propagated mutation profile” indicating proximity to genes mutated in each sample and a “propagated differential expression profile” indicating proximity to genes differentially expressed in each sample. We then use these vectors to extract descriptive features for each protein, to be used for predicting its involvement in the disease being studied.

### Summary of Results

We apply the proposed method to breast cancer (BRCA) and glioblastoma multiforme (GBM) data obtained from The Cancer Genome Atlas (TCGA) project. First, we assess the power of mutation data, expression data, and network-based integration of these two in unsupervised prediction of genes known to play a role in each cancer. We show that one can gain significant predictive power by propagating mutation or expression data over a PPI network, as compared to using raw mutation or differential expression data (area under ROC curve (AUC) gains of 0.16–0.18 for BRCA and 0.17–0.27 for GBM). We then combine the two signals to derive several features and used these features to train a supervised predictor with further improved AUC of 0.836 for BRCA and 0.933 for GBM. Importantly, by using this predictor we are able to recover important proteins that are not readily revealed by molecular data. These genes are supported by the literature and by an independent cancer gene resource. This observation suggests that incorporation of network data can provide insights beyond what can be gleaned from sequence or expression data in isolation. Seven of those novel predictions are further found be significantly predictive of patient outcome. Our results also suggest important features that contribute significantly to the prediction of causal genes in breast cancer and glioblastoma multiforme, which provide insights into how the crosstalk among mutated and differentially expressed proteins contributes to pathogenesis.

## Methods

In this section, we first describe the datasets we use. We then explain how we use network propagation for each sample to generate “propagated mutation” and “propagated differential expression” profiles for each gene. Finally, we describe the features we extract from these propagated mutation and differential expression profiles and how we use those features to develop a model to predict causal genes in cancer.

### Description of Data

The input to our method consists of BRCA (breast cancer invasive carcinoma) and GBM (glioblastoma multiforme) data obtained from TCGA [13]. We use two categories of data: somatic mutations obtained from whole-exome sequencing and microarray gene expression data. We also obtain differential expression status for TCGA samples from the COSMIC cancer gene census [2]. We collect this data into a binary mutation matrix *M*, and a binary differential gene expression matrix *D*, with samples as rows and genes as columns. We use *C*(*A*) to denote the set of column labels of matrix *A*, so that *e.g*. *C*(*M*) is the set of genes that appear in the TCGA somatic mutation data. Similarly, we define *R*(*A*) as the set of row labels of matrix *A*, corresponding to the distinct samples present in each data set.

The mutation matrices *M* are defined as

$$M\left[i,j\right]=\left\{\begin{array}{cc} 1 & \text{if}\;\text{gene}\;j\;\text{is}\;\text{mutated}\;\text{in}\;\text{sample}\;i,\\ 0 & \text{otherwise} \end{array}\right.$$ (1)

The differential expression matrices *D* are defined similarly, using differential expression status instead of somatic mutation status for each gene. BRCA data includes somatic mutations in 15189 genes across 974 samples, and differential expression in 18018 for 973 samples. GBM data likewise includes 9507 genes and 591 samples, with differential expression measurements in 17660 genes across the same 591 samples.

We use the HIPPIE protein-protein interaction network [14] (version released 2014-09-05), which contains confidence scores for 160215 interactions over 14680 proteins. All samples present in the gene expression data also appear in the mutation data. 12042 genes are contained in both the mutation and expression data, out of which 9303 are present in the HIPPIE network.

### Sample-Specific Network Propagation

We use the network propagation method described in Vanunu *et al*. [4]. Given a network *G* = (*V*, *E*, *w*) with *V* as the set of proteins, *E* as the set of their interactions, *w*(*u*, *v*) representing the reliability of an interaction *uv* ∈ *E*, and a prior knowledge vector *Y*: *V* → [0, 1], we seek to compute a function *F*(*v*) ∀*v* ∈ *V* that is both smooth over the network and accounts for the prior knowledge about each node. In the context of our problem, the prior knowledge about each node is the mutation or differential expression status of the respective gene in a sample.

As described by Vanunu *et al*. [4], we use Laplacian normalization to produce the normalized network edge weight *w*′. Briefly, we construct a |*V*| × |*V*| matrix *W* from the edge weights *w*, and construct a diagonal matrix Δ with Δ[*i*, *i*] = ∑_*j*_ *W*[*i*, *j*]. The normalized weight matrix is computed as *W*′ = Δ^−1/2^ *W*Δ^−1/2^. Our *W*′ is a 14680 × 14680 sparse matrix with each row and column corresponding to a node in the HIPPIE network, and each nonzero entry signifying an interaction between two proteins.

With the normalized weight matrix *W*′, we use the iterative procedure described by Zhou *et al*. [15] to compute *F*. Namely, starting with *F* ^(0)^ = *Y*, we update F at iteration *t* as follows:

$$F^{\left(t\right)}=\alpha W^{\prime }F^{\left(t-1\right)}+\left(1-\alpha \right)Y$$ (2)

This procedure is repeated iteratively until convergence; namely we stop the iterations when ‖*F* ^(*t*)^ − *F* ^(*t*−1)^‖_2_ < 10^−6^.

We use network propagation on a sample-specific basis to compute propagated mutation and differential expression vectors for each sample. Namely, we produce new “propagated” matrices *M*_*P*_ and *D*_*P*_, by separately using each row of matrices *M* and *D* as the prior knowledge vector *Y* in Eq 2. This is illustrated in Fig 1.

Given the data matrix *A* (either *M* or *D*) and each protein in the network *v* ∈ *V*, we construct the vector $Y_{i}^{\left(A\right)}$ for sample *i* as follows:

$$Y_{i}^{\left(A\right)}\left[v\right]=\left\{\begin{array}{cc} A[i,v] & \text{if}\;v\in C(A)\cap V,\\ 0 & \text{otherwise} \end{array}\right.$$ (3)

That is, the prior knowledge about a protein is 1 if and only if the protein is part of the HIPPIE network and the corresponding gene is mutated in sample *i* or differentially expressed in it. For each sample *i* ∈ *R*(*A*), we denote the prior information vectors by $Y_{i}^{\left(M\right)}$ and $Y_{i}^{\left(D\right)}$. Subsequently, using each of these prior information vectors, we use the iterative procedure described above to compute propagated mutation and expression vectors, denoted respectively as $F_{i}^{\left(M\right)}$ and $F_{i}^{\left(D\right)}$ for sample *i*.

Next, we collect each propagated vector $F_{i}^{\left(A\right)}$ into the rows of a “propagated” matrix *A*_*P*_, where *R*(*A*_*P*_) = *R*(*A*) and *C*(*A*_*P*_) = *V*. Intuitively, the propagated matrices *M*_*P*_ and *D*_*P*_ contain the per-sample binary vectors of *M* and *D* smoothed over the network. In biological terms, each row of these matrices represents the network proximity of each gene product to mutated and differentially expressed genes in that sample. Consequently, as illustrated in Fig 1, the columns of these matrices provide propagated mutation and differential expression profiles for each gene product across all samples, indicating the proximity of the respective gene product to the products of mutated or differentially expressed genes in the respective sample.

### Consolidation of Mutation and Expression Data

We seek to use the propagated mutation and differential gene expression matrices *M*_*P*_ and *D*_*P*_ (with sample set *S* = *R*(*M*_*P*_) = *R*(*D*_*P*_)) to predict causal genes based on network proximity to mutated and differentially expressed genes in BRCA. To this end, we define several features that express the mean, variance and cross-correlation of the columns of those matrices across the *n* = |*S*| samples:

1. $\mu_{M}\left[g\right]=\frac{1}{n}\sum_{i}^{n}M\left[i,g\right]$: mutation frequency of gene *g* across samples.
2. $\mu_{M_{P}}\left[g\right]=\frac{1}{n}\sum_{i}^{n}M_{P}\left[i,g\right]$: mean of propagated mutation scores across samples. *μ*_*M*_*P*__[*g*] quantifies the mean proximity of gene *g* to mutated genes across all samples.
3. $\sigma_{M_{P}}^{2}\left[g\right]=\mathrm{Var}M_{P}\left[\cdot ,g\right]$: variance of propagated mutation scores across samples. $\sigma_{M_{P}}^{2}\left[g\right]$ quantifies how inconsistently the gene products in the neighborhood of gene *g* are mutated across different samples.
4. $\mu_{D}\left[g\right]=\frac{1}{n}\sum_{i}^{n}D\left[i,g\right]$: differential expression frequency across the *n* samples.
5. $\mu_{D_{P}}\left[g\right]=\frac{1}{n}\sum_{i}^{n}D_{P}\left[i,g\right]$: mean of propagated differential expression scores across the *n* samples. *μ*_*D*_[*g*] quantifies the mean proximity of gene *g* to differentially expressed genes across all samples.
6. $\sigma_{D_{P}}^{2}\left[g\right]=\mathrm{Var}D_{P}\left[\cdot ,g\right]$: variance of propagated differential expression scores across samples. $\sigma_{D_{P}}^{2}\left[g\right]$ quantifies how inconsistently the gene products in the neighborhood of gene *g* are differentially expressed across different samples.
7. *ρ*[*g*] = Spearman correlation between *M*_*P*_[·, *g*] and *D*_*P*_ [·, *g*]. *ρ*[*g*] quantifies whether samples that harbor mutations in the neighborhood of gene *g* also harbor differentially expressed genes in the neighborhood of gene *g* and vice versa.
8. $\delta \left[g\right]=\sum_{i}^{n}M_{P}\left[i,g\right]\cdot D_{P}\left[i,g\right]$: dot product between *M*_*P*_[⋅, *g*] and *D*_*P*_[⋅, *g*]. *δ*[*g*] can be interpreted similarly as *ρ*[*g*]. However, unlike correlation, this is a non-normalized measure of the consistency of proximity to mutated and differentially expressed genes. As such, *δ*[*g*] includes information about the magnitude of values in columns *M*_*P*_[⋅, *g*] and *D*_*P*_[⋅, *g*] as well as the agreement between those columns.
9. *χ*_max_[*g*] and *χ*_mean_[*g*]: For a gene *g*, high *χ*[*g*] scores denote a gene that is in close proximity to other genes that are frequently mutated *or* frequently differentially expressed.
  - *χ*_max_[*g*] = max_*i* ∈ *S*_(max{*M*_*P*_[*i*, *g*], *D*_*P*_[*i*, *g*]}). A high *χ*_max_[*g*] denotes a gene that is close to mutations or differential expression in any patient.
  - $\chi_{\mathrm{mean}}\left[g\right]=\frac{1}{n}\sum_{i}^{n}\max\{M_{P}\left[i,g\right],D_{P}\left[i,g\right]\}$. *χ*_mean_[*g*] represents the gene’s mean distance to mutations or differential expression across all samples.
10. *ν*_max_[*g*], *ν*_mean_[*g*]: A high *ν*[*g*] score denotes a gene that is in close proximity to other genes that are frequently mutated *and* frequently differentially expressed.
  - *ν*_max_[*g*] = max_*i* ∈ *S*_(min{*M*_*P*_[*i*, *g*], *D*_*P*_[*i*, *g*]}). A high *ν*_max_[*g*] denotes a gene that is close to mutations and differential expression in any sample.
  - $\nu_{\mathrm{mean}}\left[g\right]=\frac{1}{n}\sum_{i}^{n}\min\{M_{P}\left[i,g\right],D_{P}\left[i,g\right]\}$. *ν*_mean_[*g*] quantifies the gene *g*’s mean distance to mutations and differential expression across all samples.
11. *γ*[*g*]: Network centrality of gene *g*, as quantified using eigenvector centrality. Propagation of mutation and differential expression data across the network may bias results in favor of nodes that are central to the network or have high degree [3]. Our propagation method uses node degrees to normalize edge weights, offering some correction for nodecentrality [4]. However, to explicitly account for node centrality without unfairly penalizing hub nodes, and to gain insights into the effect of network centrality, we include network centrality as a feature in the model.

**Fig 2:**
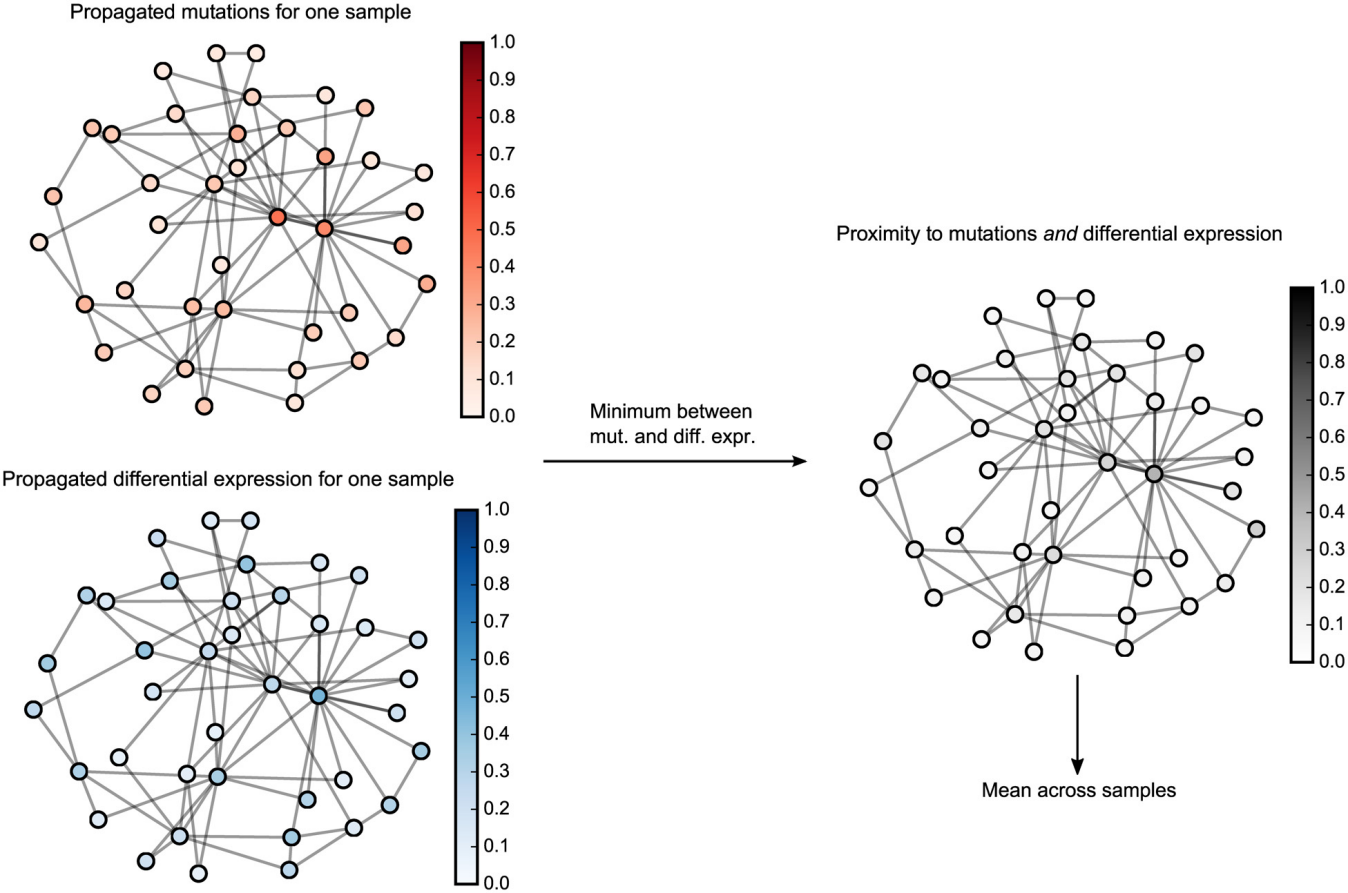
Visualization of feature *ν*_mean_ across a simulated data set with three samples and mutations across 40 genes.

An example of the *ν*_mean_ feature in a simulated data set is shown in Fig 2. We see that genes which score highly via propagated mutation and differential expression frequency are scored highly with *ν*_mean_, conversely, genes that are proximal to only mutations *or* differential expression may be scored highly in each individual data set but need not be scored highly in this combined feature.

The features described above are used as input to a standard logistic regression model to predict the causal status of gene *g*. To train this model, we use prior knowledge of whether each gene is known to be associated with breast cancer based on the integrated breast cancer pathway (Table A in S1 Text), or in glioblastoma based on the GBM KEGG pathway (Table B in S1 Text). The logistic regression model represents the probability *p* that a gene is associated with the cancer of interest as

$$\log\frac{p}{1-p}=\beta_{0}+\beta_{1}x_{1}+\ldots +\beta_{n}x_{n}.$$ (4)

Here, *β*_0_ represents the background probability that a gene is related to the disease, each *x*_*i*_ represents one of the features described above, and each *β*_*i*_ represents the magnitude to which *x*_*i*_ influences *p*. In addition to estimating the magnitude of a feature’s effect on *p*, logistic regression models also allow for the investigation of whether a feature is *statistically* significant in the model fit. This framework therefore allows us to examine the relationship between the role of a gene in cancer and its mutational frequency, differential expression frequency, network distance to mutations or differential expression, and the relationship between these distances.

Using the genes labeled based on prior knowledge of the molecular basis of each cancer, we fit this model using the features described above, perform step-down via AIC (Akaike Information Criterion [16]), and use the probabilistic output of the stepped-down model as prediction scores for further analysis. We perform experiments to investigate whether this model can effectively recover cancer-related genes even though they are not frequently mutated or differentially expressed in available samples. We also evaluate the model’s performance on an independently curated set of genes known to be implicated in cancer. Finally, we investigate which features significantly contribute to the model fit, in order to gain insights into the factors that have important roles in pathogenesis.

## Results/Discussion

In this section, we apply the logistic regression model we have trained to predict genes associated with breast cancer and glioblastoma and evaluate its performance and the contribution of the different features to its success. Subsequently, we examine in detail the novel predictions made by our model. We identify several predictions that are supported by the literature and find that our predictions significantly overlap with an independent resource on cancer genes. Finally, we test the clinical relevance of the predicted genes, identifying several promising candidates with significant predictive power with respect to patient survival.

### Recovering Known Cancer Genes

We evaluate the predictive ability of our model using ROC curves, using the integrated breast cancer pathway from the NCBI BioSystems database [17] and the glioblastoma KEGG pathway [18]. We label a gene as positive if and only if it is contained in the respective pathway, and use these positive/negative labels to evaluate various prediction schemes. Better scoring systems naturally induce a higher area under the ROC curve (AUC).

**Fig 3:**
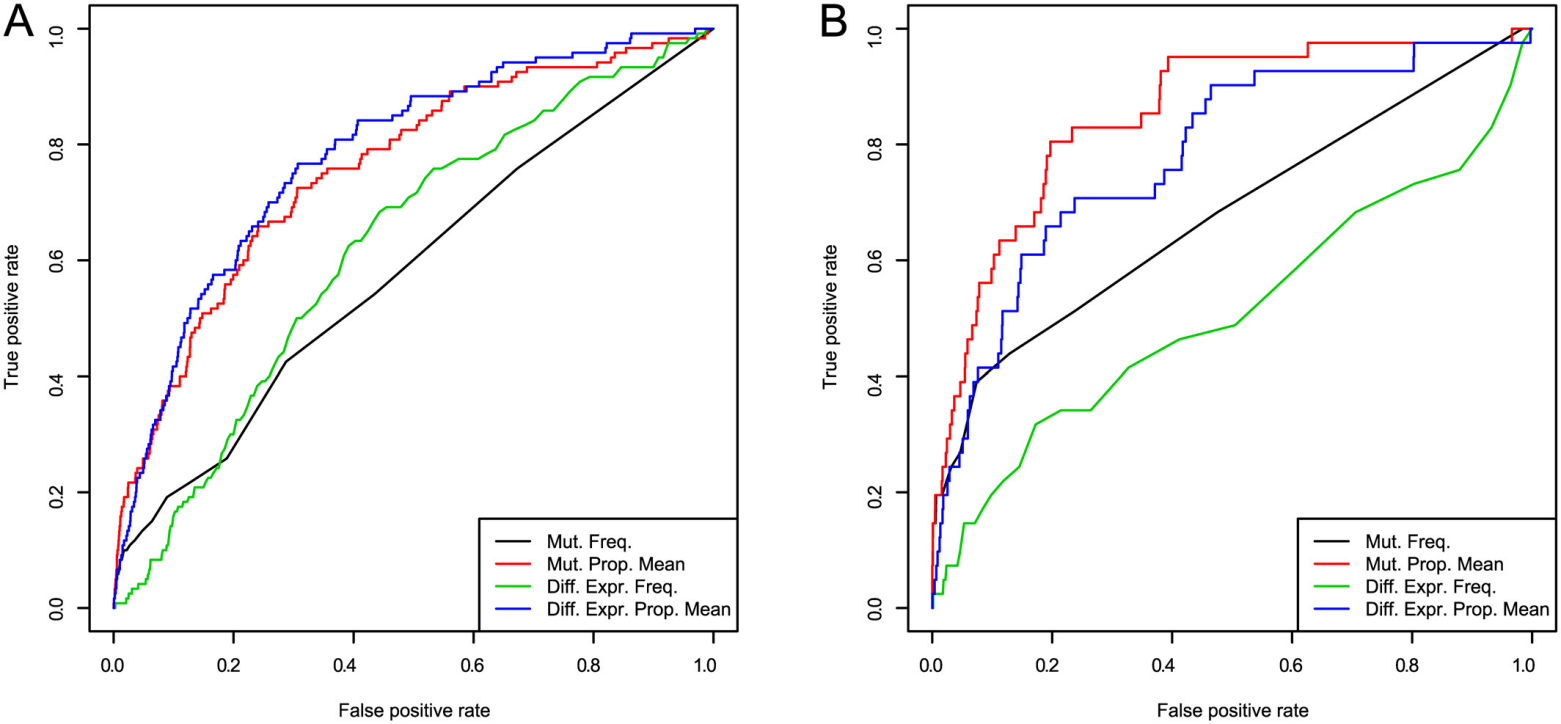
ROC curves for BRCA (a) and GBM (b) for single scoring methods: mutation frequency, differential expression frequency, and column means *μ*_*M*_ and *μ*_*G*_ of the matrices *M*_*P*_ and *D*_*P*_, respectively.

We first examine the ability of naïve scoring methods in recovering known BRCA and GBM genes. Namely, we investigate how each of mutation frequency, differential expression frequency, and the network propagated mutation and differential expression, *i.e*., respectively the column-wise means of matrices *M*, *D*_*G*_, *M*_*P*_ and *D*_*P*_ described in “Consolidation of Mutation and Expression Data”, can predict known BRCA and GBM genes. The results of this analysis are shown in Fig 3 and Tables 1 and 2. We see that both mutation and differential expression frequency are slightly informative (AUC 0.581 and 0.625, respectively) in choosing genes that are part of the integrated BRCA pathway. In other words, frequency of mutation or differential expression in TCGA breast cancer samples provides some information on whether a gene is involved in the BRCA pathway, but this information is quite modest.

**Table 1:** AUC values for BRCA for single scoring methods: mutation frequency, differential expression frequency, and column means *μ*_*M*_ and *μ*_*G*_ of the matrices *M*_*P*_ and *D*_*P*_, respectively.

**Score**	**AUC**
Mut. Freq	0.581
Mut. Prop. Mean	0.757
Diff. Expr. Freq.	0.625
Diff. Expr. Prop. Mean	0.781

**Table 2:** AUC values for GBM for single scoring methods: mutation frequency, differential expression frequency, and column means *μ*_*M*_ and *μ*_*G*_ of the matrices *M*_*P*_ and *D*_*P*_, respectively.

**Score**	**AUC**
Mut. Freq	0.679
Mut. Prop. Mean	0.854
Diff. Expr. Freq.	0.511
Diff. Expr. Prop. Mean	0.782

We see that the propagated signals (with propagation parameter *α* = 0.8) show much more discriminative power: the mutational AUC increases to 0.757 after network propagation, and likewise the differential expression AUC increases to 0.781. We see similar gains in predictive power in GBM: raw mutational and differential expression AUC are informative (AUC 0.679 and 0.511, respectively), and the application of network propagation to these signals boots the AUC values to 0.854 and 0.782.

Though the increase in predictive power through network propagation is considerable, we seek to improve the AUC values further through a more sophisticated integration of the propagated mutation and differential expression signals. For this purpose, we evaluate the regression model described in subsection “Consolidation of Mutation and Expression Data.”

**Fig 4:**
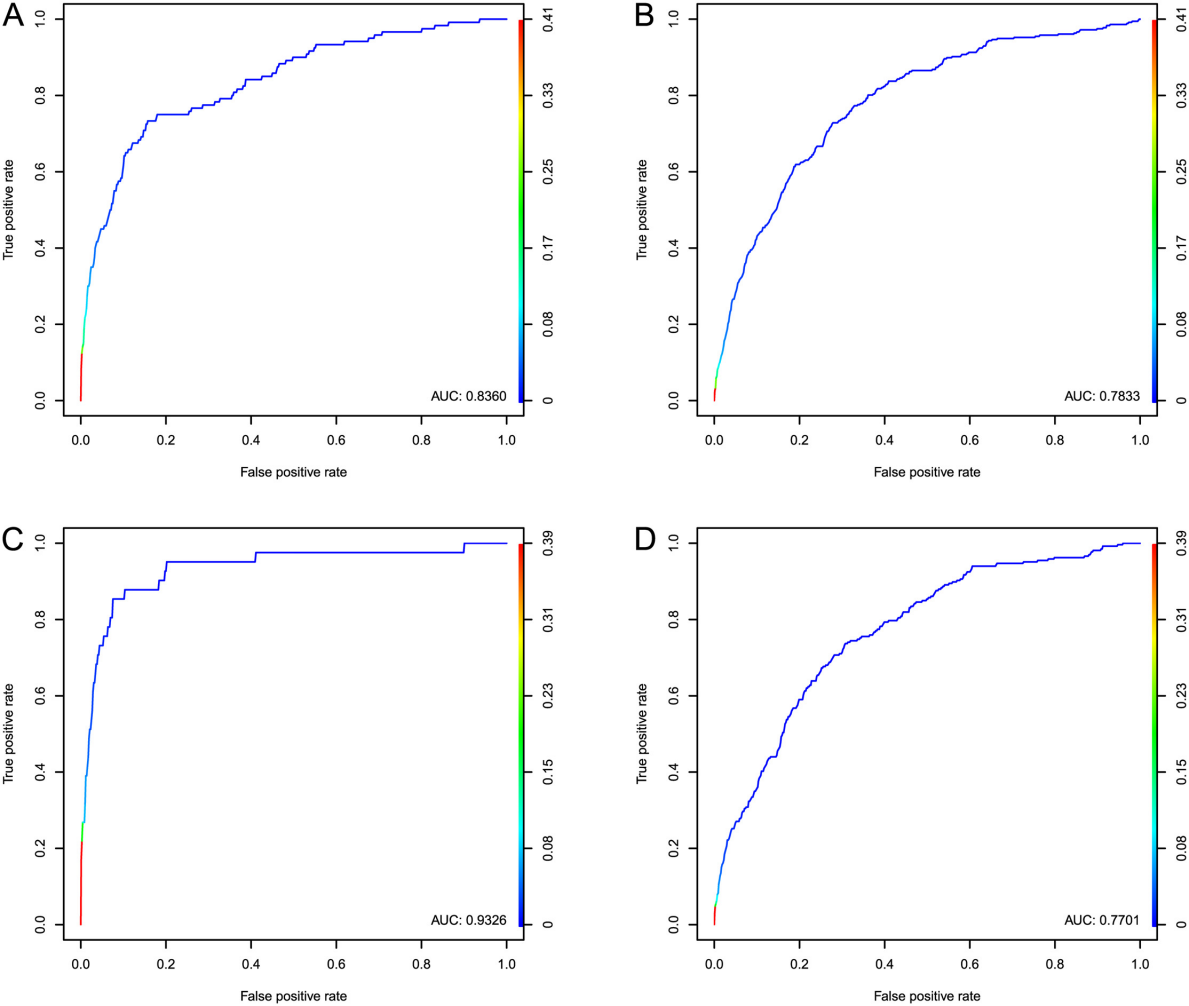
ROC curves of predictions from the stepped-down models described in Tables 3 and 4. (a) shows genes contained in the integrated BRCA pathway, (c) shows genes contained in the GBM KEGG pathway, (b) and (d) show prediction of causal genes on an independent dataset: the COSMIC cancer gene census. Color bars on the right axes denote thresholds on the prediction score; the color along each ROC curve shows the true and false positive rate at each threshold value.

We first fit the logistic regression model described in the aforementioned section to the full data sets, and perform a step-down procedure to remove features that do not significantly contribute to the model fit. We use the standard AIC (Akaike information criterion) measure [16] to determine whether a model term should be preserved. At each iteration of the step-down procedure, the AIC is computed for the full model and for reduced models with each single term removed. The term whose removal most improves AIC is removed from the model. The step-down procedure terminates when no term removal improves AIC. Fig 4 shows ROC curves resulting from this analysis; Fig 4a and 4c respectively show performance in recovering genes in the BRCA and GBM pathways. Fig 4b shows the accuracy in predicting genes’ membership in the COSMIC database using the BRCA model, and likewise Fig 4d shows performance in predicting COSMIC membership using the model trained from GBM data. We see that the stepped-down models improve ROC AUC when compared to the single features shown in Fig 3, and perform well when selecting genes contained in the COSMIC set.

### Evaluation of Features

The final model coefficients and *P*-values for each disease are shown in Tables 3 and 4. For BRCA, we see that *ν*_mean_ and *δ* are highly significant predictors of a gene’s membership in the integrated BRCA pathway, with a positive coefficient for *ν*_mean_ and a negative coefficient for *δ*. We also see a large negative coefficient for feature *χ*_mean_. We interpret this result by noting that for some sample *i* and gene *j*, the value max{*M*_*P*_[*i*, *j*], *D*_*P*_[*i*, *j*]} is high if gene *j* is close to either mutations *or* differential expression, and genes that score highly in only one of these measures are likely to simply be frequently mutated or differentially expressed. Conversely, the *ν* signals measure the degree to which a gene is close to *both* mutations and differential expression. We indeed see that *ν*_mean_ is significant (*P* < 2 × 10^−16^) with positive coefficient 91.7. We see similar trends in GBM: again *ν*_mean_ is the most significant individual feature, with positive coefficient, and *δ* is also significant with a negative coefficient. Unlike BRCA, in GBM the *χ* features which select for proximity to mutations *or* differential expression are not preserved after AIC step-down. It is also notable that *δ* is preserved in both diseases but *ρ* is not. This result is not entirely surprising since *ρ* only represents agreement between propagated differential expression and mutation signals, and *δ* also quantifies a gene’s *total* proximity to mutations and differential expression.

**Table 3:** Logistic regression coefficients and *P*-values for the stepped-down model described in subsection “Recovering Known Cancer Genes” for BRCA.

**Feature**	**Estimate**	***P*-value**
Intercept	−6.7388	< 2 × 10^−16^
*μ*_*D*_*P*__	615.7567	0.000190
*δ*	−139.1464	1.58 × 10^−5^
*χ*_mean_	−611.9088	0.000199
*ν*_max_	−1.7107	0.057922
*ν*_mean_	91.7271	< 2 × 10^−16^
*μ*_*M*_	124.3517	0.009907
$\sigma_{M_{P}}^{2}$	50.5214	0.046352

**Table 4:** Logistic regression coefficients and *P*-values for the stepped-down model described in subsection “Recovering Known Cancer Genes” for GBM.

**Feature**	**Estimate**	***P*-value**
Intercept	−7.210	< 2 × 10^−16^
*μ*_*D*_	21.506	0.000625
$\sigma_{D_{P}}^{2}$	−26.777	0.001136
*δ*	−112.026	9.34 × 10^−5^
*ν*_max_	−2.564	0.192329
*ν*_mean_	154.710	< 2 × 10^−16^
*μ*_*M*_	−25.330	0.003619
$\sigma_{M_{P}}^{2}$	24.319	0.000748

**Fig 5:**
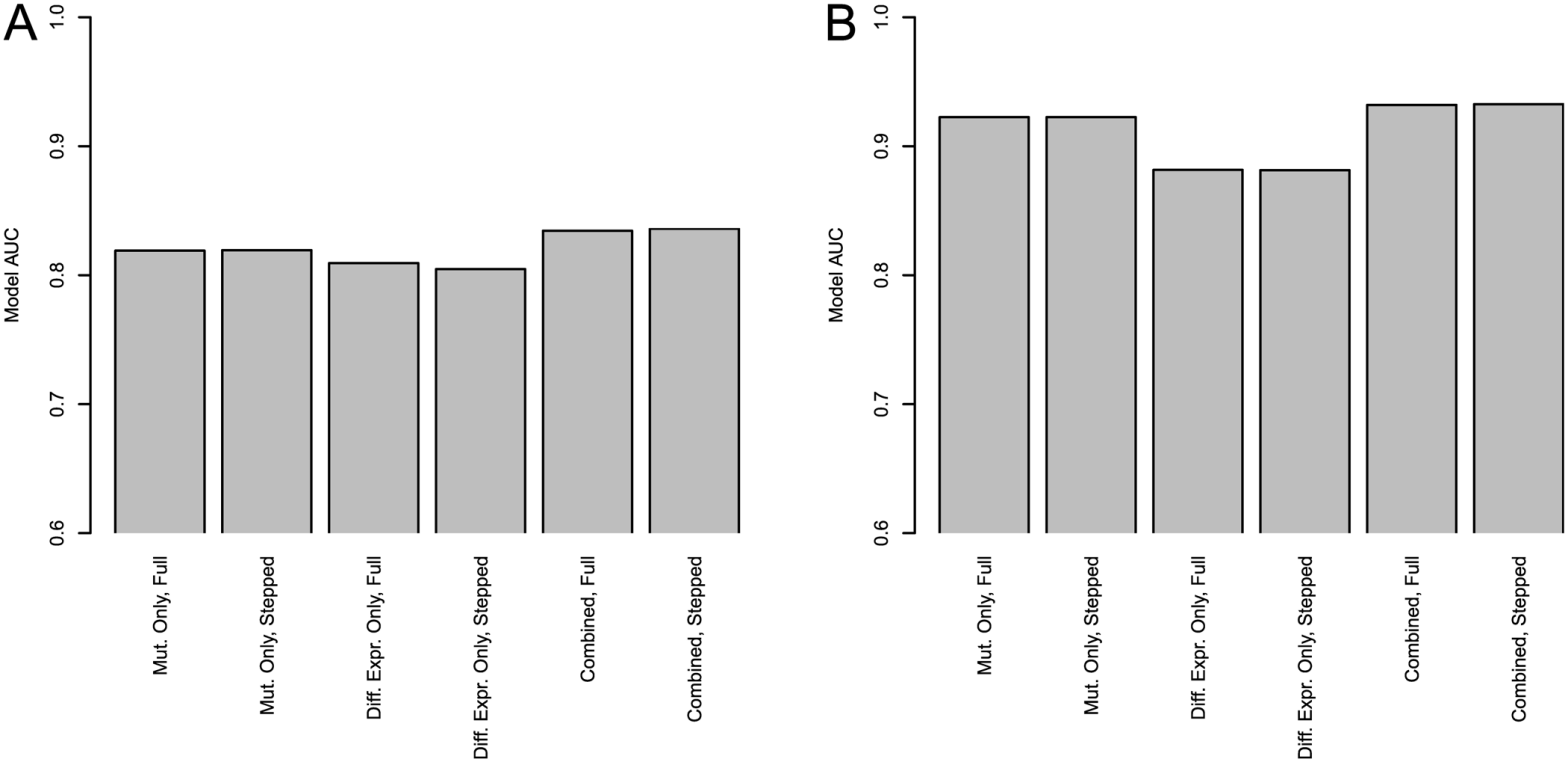
ROC AUC values for predictive models fit with subsets of available features. (a) shows BRCA, (b) shows GBM.

We also evaluate the predictive power added by our combined features in comparison to models fit with purely mutational and differential expression data. These results are shown in Fig 5. These results show ROC AUC values for six models in each disease: one fit with all available mutational features, one fit with all available differential expression features, the full model with all features, and stepped-down versions of the three aforementioend models. We see that in both BRCA (Fig 5a) and GBM (Fig 5b), the combined models improve on performance of those fit with only mutational or differential expression features.

**Fig 6:**
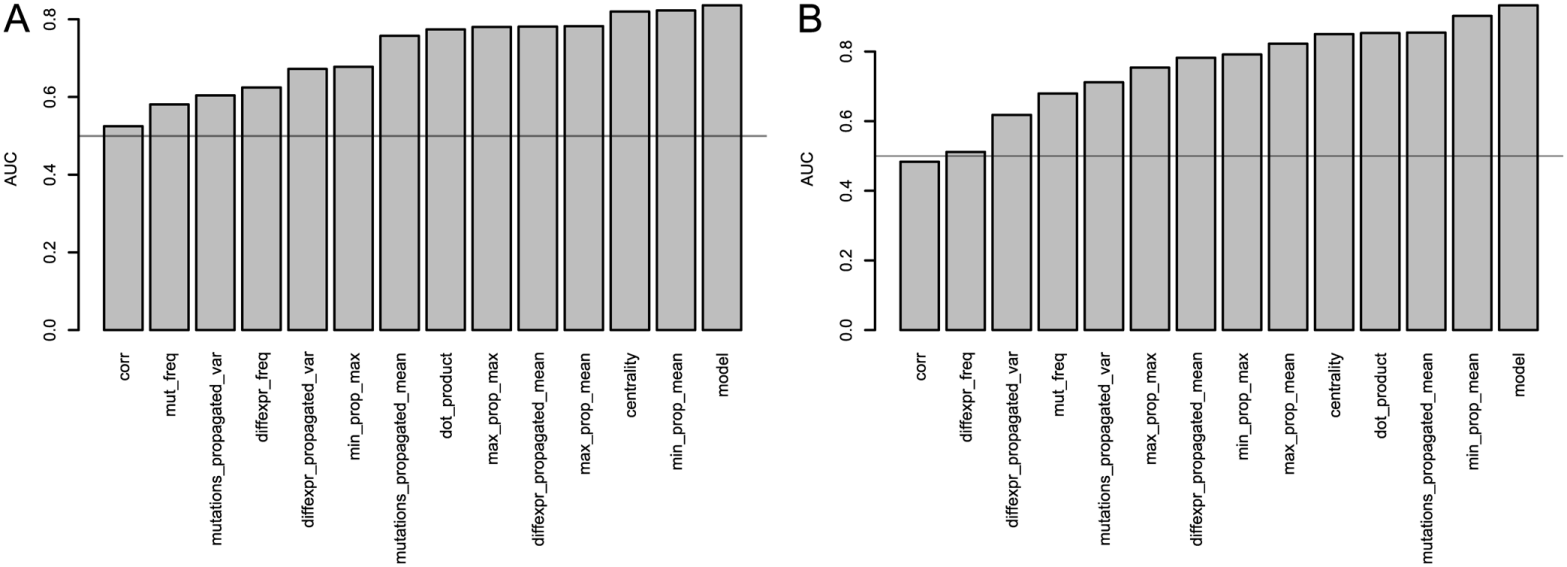
AUC scores of univariate predictors included in the BRCA and GBM models, in comparison with the AUC scores of the models themselves. (a) shows BRCA, (b) shows GBM.

Additionally, we evaluate the distribution and univariate predictive power of each individual feature included in the predictive models shown above. Fig 6 shows the AUC values for each feature defined in “Consolidation of Mutation and Expression Data” in comparison with the AUC value of the fitted model that combines individual features. Fig 6a shows the AUC values for recovering BRCA genes; Fig 6b shows GBM. In both cases we see that *ν*_mean_ is the most informative individual feature, which favors genes that are close to both mutations and differential expression. In both BRCA and GBM we see that the predictive model improves upon the AUC values of each individual predictor.

We observe that the mean propagated mutation feature (*μ*_*M*_*P*__) provides better predictive performance than mutation frequency (*μ*_*M*_) for BRCA. However, this feature is dropped from the stepped down model while mutation frequency is preserved. This observation applies to several other features for both BRCA and GBM as well. This observation demonstrates the benefit of using logistic regression, in that features that are themselves significant may be almost colinear and not all of them need to be preserved if there is overlap in the information provided by multiple features. In particular, the specific observation stated above suggests that the smoothed mutational signal in *μ*_*M*_*P*__ is subsumed by the combined features, whereas mutation frequency provides information in addition to the information provided by other selected features. It is also interesting to note that the coefficient of mutation frequency is negative in the stepped down model. It is likely that this reflects a correction for passenger mutations (mutated genes that are not functionally related to tumorigenesis), since the information provided by driver mutations (mutated genes that play a role in tumorigenesis) is incorporated by another feature (combined propagated mutation and differential expression signals) in the model.

**Fig 7:**
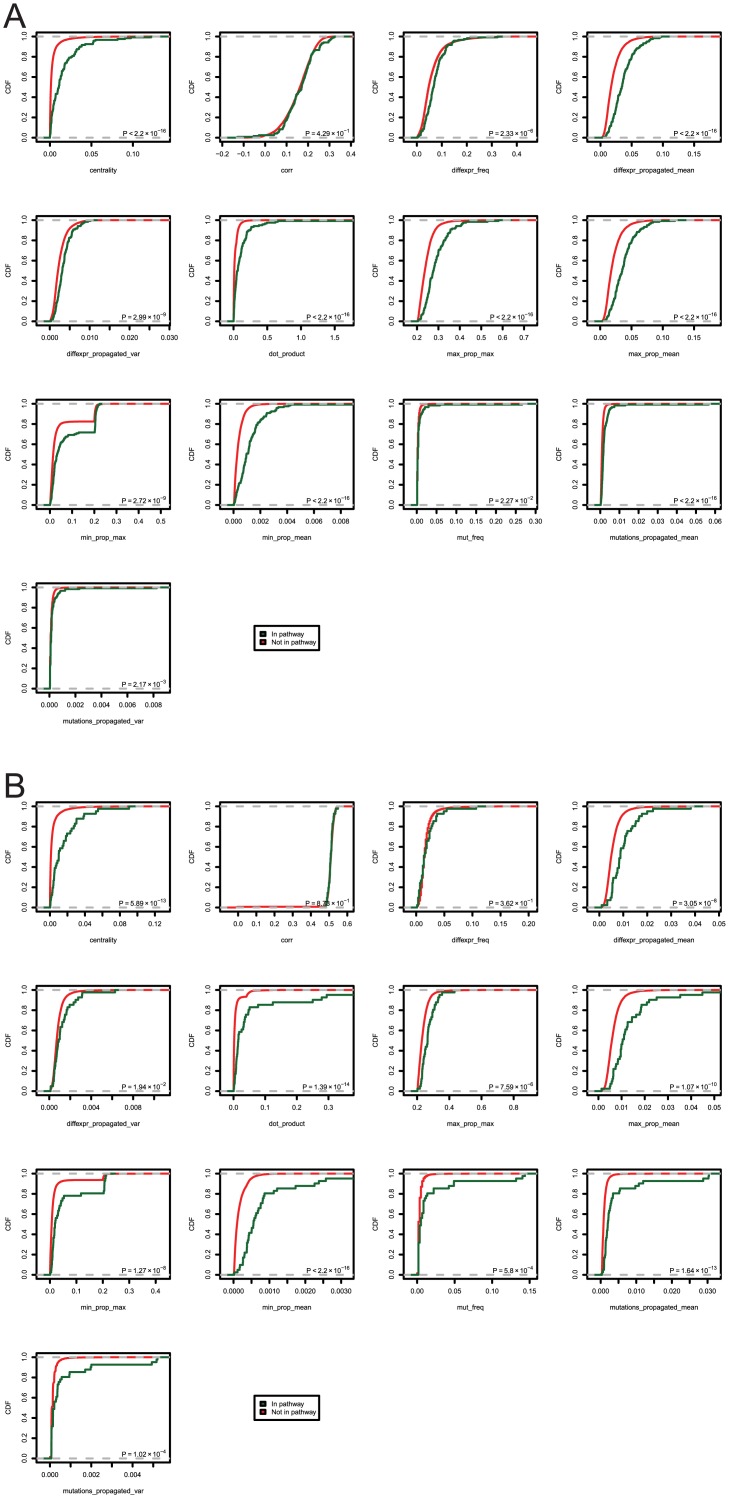
CDF curves for individual features included in the prediction model. *P*-values show Kolmogorov-Smirnov test results. Genes are separated by pathway membership; (a) shows BRCA, (b) shows GBM.

Fig 7 shows the CDFs of each individual feature, with separate curves for genes that are contained in each respective pathway. Fig 7a showsBRCA; Fig 7b shows GBM. These figures indicate significant difference between cancer genes and other genes in terms of the distribution of some individual features, and reveal bimodality in *δ* (dot product) in GBM and *ν*_max_ (minimum between *M*_*P*_ and *D*_*P*_, maximum across samples) in both diseases.

**Fig 8:**
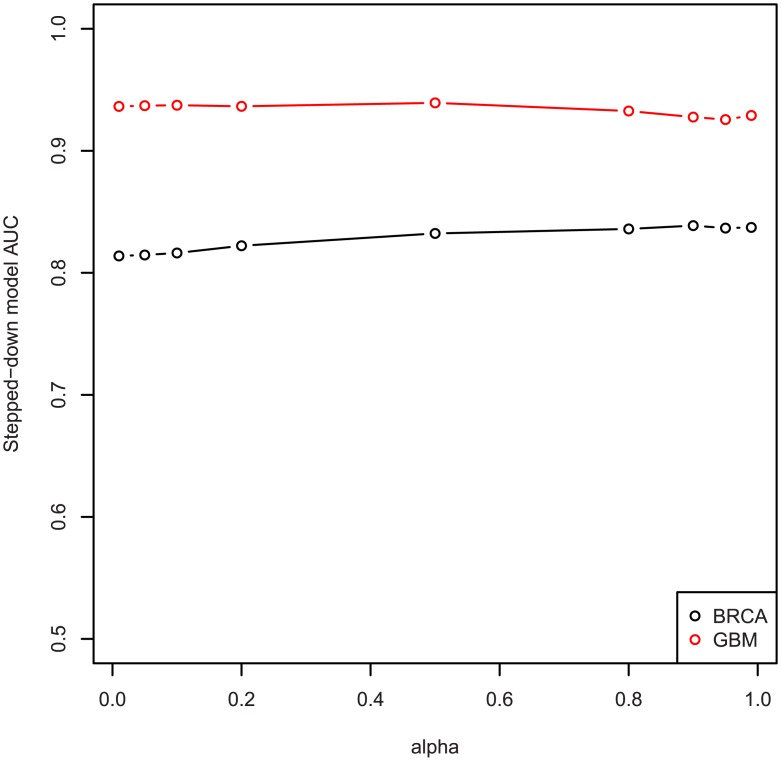
AUC scores of predictive models fit with varying *α*, for *α* ∈ {0.01, 0.05, 0.1, 0.2, 0.5, 0.8, 0.9, 0.95, 0.99}.

We also fit models with multiple values of the propagation parameter *α*, ranging from 0.01 to 0.99. The results are shown in Fig 8, and we see that the performance of stepped-down predictive models does not significantly depend on the propagation parameter *α*.

### Prediction of New Cancer Genes

**Fig 9:**
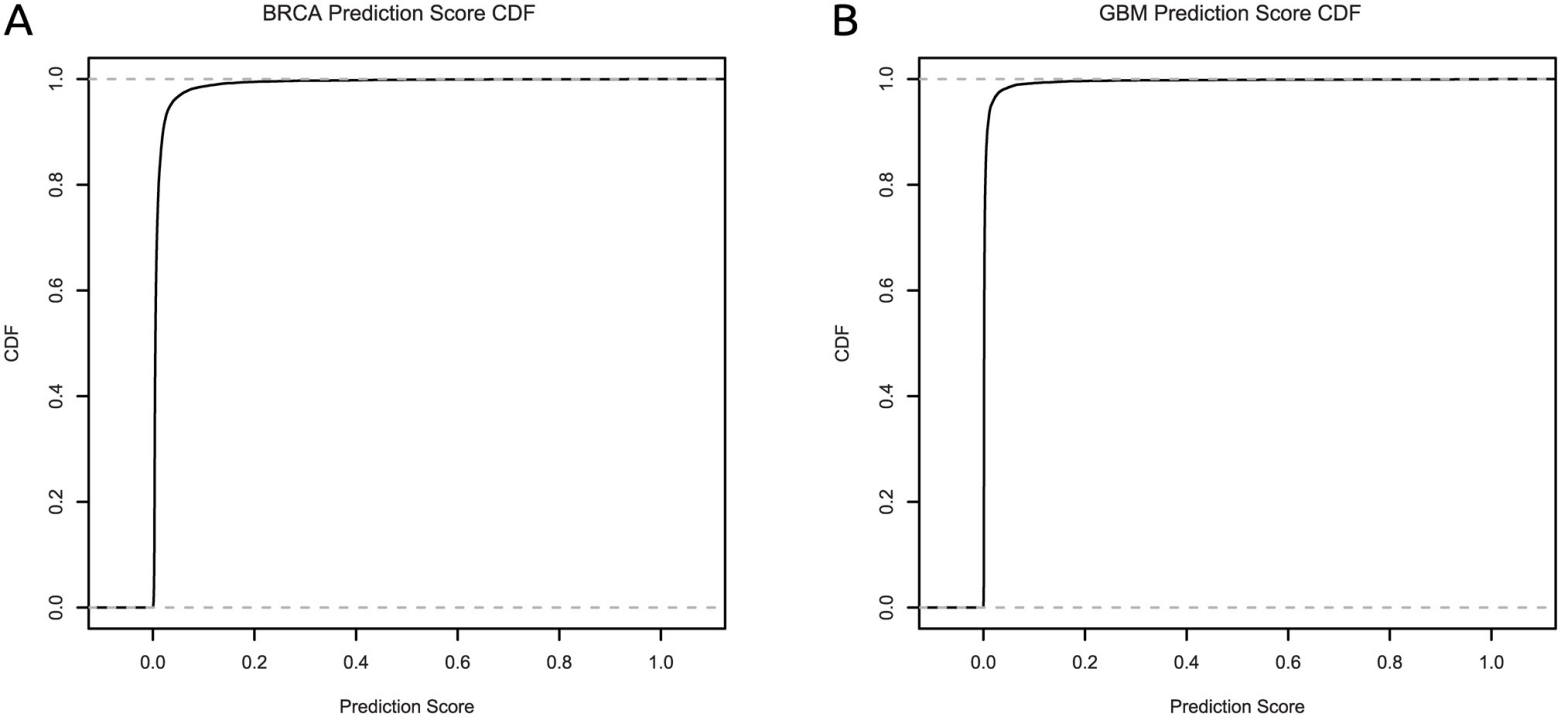
CDF curves of prediction scores from stepped-down logistic regression models for each data set. (a) shows BRCA, (b) shows GBM.

**Fig 10:**
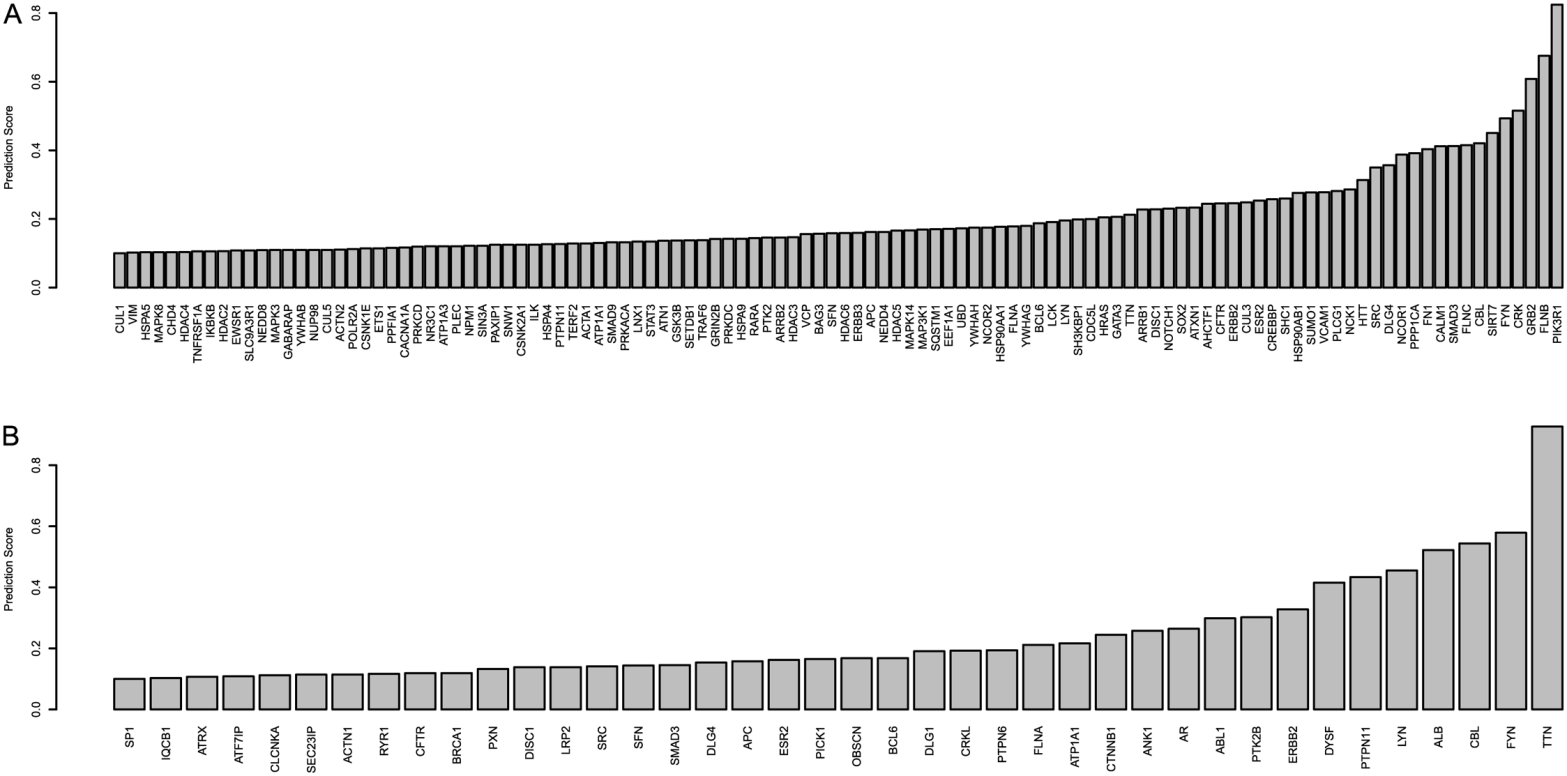
Prediction scores of highest-scoring genes that are not contained in respective pathways: BRCA in (a) and GBM in (b).

In order to evaluate the utility of our method in predicting new causal genes, we investigate the high-scoring genes that are not already known to be implicated with breast cancer and glioblastoma. The cumulative distributions of genes’ prediction scores (outputs of the stepped-down logistic regression models) are shown in Fig 9. We see that the distributions of scores are skewed toward 0, and for demonstration purposes we consider a gene to be high-scoring if its prediction score is ≥ 0.2. The highest-scoring such genes are shown along the horizontal axis of Fig 10; (Fig 10a) shows BRCA and (Fig 10b) shows GBM. Several interesting genes appear; *PIK3R1* is known to be implicated in human immunodeficiency [19] and the PI3K kinase has been shown to regulate insulin-induced cell proliferation in the MCF-7 breast cancer cell line [20]. *GRB2* interacts with *BCAR1* as part of the CIN85 complex [21], and *CBL* is a known oncogene in myeloid malignancies [22].

Since our goal is the identification of potential “silent players” that cannot be selected by each data set in isolation, we identify genes scored highly (prediction score ≥ 0.2) by the combined model (Tables 3 and 4) that are not scored highly by the models shown in Fig 5. Genes for BRCA are shown in Table 5 and genes for GBM are shown in Table 6. Many of these genes are known to be implicated in diseases, but few have been previously reported as associated with cancer. *GATA3* controls differentiation of luminal cells in mammary glands [23]. *HRAS* mutations have been reported to cause altered glucose metabolism in mammary carcinogenesis [24] and to promote epithelial-mesenchymal transition in mammary epithelial cells [25]. *NOTCH1* [26] has previously been associated with head and neck squamous cell carcinoma [27], acute lymphoblastic leukemia [28], and chronic lymphocytic leukemia [29]. *SHC1* interacts with the atypical kinase *PEAK1*, which is involved in a basal breast cancer signaling pathway [30]. Alterations in methylation of *ANK1* are common in Alzheimer’s disease [31, 32]. Overexpression of *ERBB2* (also known as *HER2*) has been shown in several cancers, including non-small cell lung [33] and endometrial cancers [34]. Mutations in the tyrosine phosphatase *PTPN11* have been shown to cause a predisposition for leukemia and some solid tumors [35].

**Table 5:** Potential “silent players” in BRCA identified by the combined model shown in Table 3: genes scored highly by the stepped-down model fit with combined features, that do not score highly in models fit with only mutational or differential expression features.

**Gene**	**Prediction Score**
*AHCTF1*	0.244284
*ARRB1*	0.227461
*ATXN1*	0.233371
*CFTR*	0.245543
*DISC1*	0.228234
*ERBB2*	0.246014
*FLNC*	0.415142
*GATA3*	0.206499
*HRAS*	0.204533
*NCOR1*	0.387477
*NOTCH1*	0.230020
*PLCG1*	0.281162
*SHC1*	0.259797

**Table 6:** Potential “silent players” in GBM identified by the combined model shown in Table 4: genes scored highly by the stepped-down model fit with combined features, that do not score highly in models fit with only mutational or differential expression features.

**Gene**	**Prediction Score**
*ANK1*	0.257832
*ATP1A1*	0.216836
*CTNNB1*	0.244790
*DYSF*	0.415453
*ERBB2*	0.327729
*FLNA*	0.211497
*LYN*	0.455178
*PTK2B*	0.302009
*PTPN11*	0.433726

As an independent evaluation of our method, we also examine our scoring system’s ability to select genes that are included in the COSMIC cancer gene census [2]. As with our original set of BRCA interesting genes, we treat membership in the COSMIC data as a positive label for a gene, and evaluate our ability to rank these genes higher than others. Fig 4b and 4d show ROC curves for this gene selection using the models shown in Tables 3 and 4, with AUC values of 0.7833 for BRCA and 0.7701 for GBM. We evaluate the statistical significance of selection of genes in the COSMIC database among those not contained in the respective pathways for BRCA and GBM using hypergeometric tests. In BRCA, 321 genes remain in the COSMIC set after removing those that are included in the integrated BRCA pathway. 8 of the 36 genes with prediction scores ≥ 0.2 overlap with the COSMIC dataset; choosing at least 8 of 321 in 36 trials from the remaining 14562 genes yields *P* = 5.09 × 10^−8^. In GBM, 250 genes remain in the COSMIC set after removing those that are included in the respective KEGG pathway. 10 of the 40 genes with prediction scores ≥ 0.2 overlap with the COSMIC dataset; choosing at least 10 of 250 in 40 trials from the remaining 14562 genes yields *P* = 2.06 × 10^−9^.

### Association with Patient Outcome

**Fig 11:**
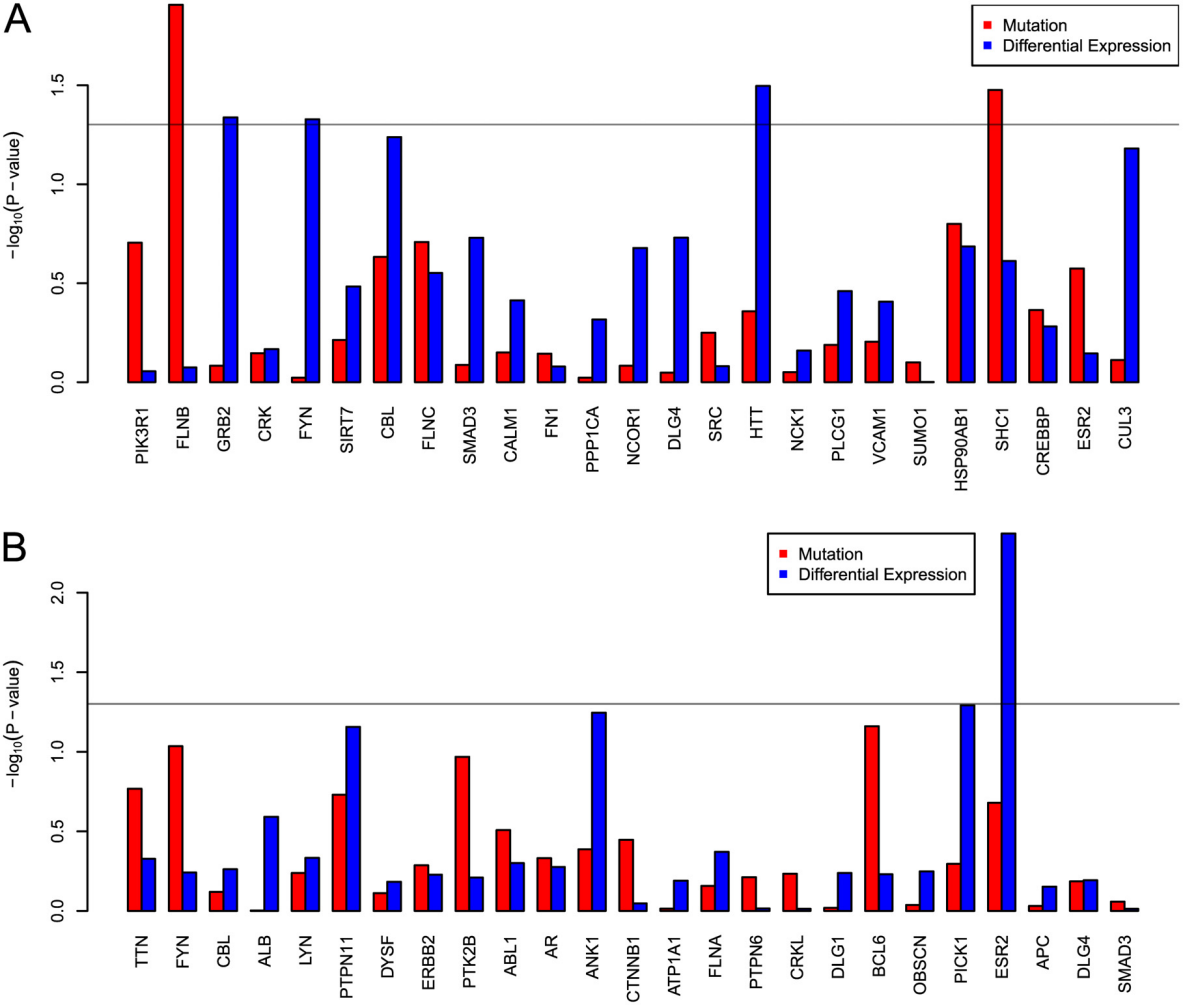
Log-rank *P*-values of differences in patient outcome (survival), using top-scoring genes that are not present each disease’s respective pathway. BRCA is shown in (a); GBM is shown in (b). For each gene, distinct tests are performed using mutation and differential expression status to separate the samples into two groups. −log_10_(log-rank *P*-value) is plotted on the *y*-axis. The horizontal grey line denotes the 0.05 *P*-value cutoff.

We also examine our method’s ability to recover genes for which mutation or differential expression status is predictive of patient outcome (survival). While the main objective of this study is not to identify markers for predicting patient outcome, these results are presented as an additional validation of the silent players we identify. As such, for both BRCA and GBM, we identify the 25 top-scoring genes that are not contained in the respective pathway, and use the mutational and differential expression status of these genes to repeatedly separate the sample set into two groups. We then use the logrank test to estimate the significance of the difference in survival between those groups; *P*-values are shown in Fig 11. BRCA samples are shown in Fig 11a, and we see nominal statistical significance from somatic mutations in *FLNB* and *SHC1*. *FLNB* is involved in vascular repair and has not been shown to be associated with cancer, but *SHC1* interacts with a kinase signaling pathway that has been implicated in breast cancer [36, 37]. Differential expression status in *GRB2*, *FYN*, and *HTT* also show utility in predicting differences in survival between groups. In GBM, we see that differential expression status of *ESR2* is also nominally significant in stratifying patient survival.

### Conclusions

Molecular data is a gold-mine for studying human disease, but current methods do not seem to exploit its full potential due to computational problems and lack of statistical power to examine all genomic markers or combinations of those. Network-based analyses provide an appealing bypass as they greatly narrow the search space. Here we have shown the power of network propagation in exploiting weak signals, from either sequence or expression studies, to predict disease causing genes. An application of our approach to breast cancer and GBM data revealed novel genes with literature support and significant association to disease outcome.

Our preliminary results can be extended in several ways. While our analysis focused on breast cancer, the methodology is general and could be applied to any multi-factorial disease for which there are available gene expression and/or sequence data. Furthermore, the method is extensible to other types of omics data such as protein expression and DNA methylation. Finally, it is interesting to study how the method can benefit from prior knowledge on disease causing genes, potentially better guiding the propagation process.

## Supporting Information

S1 Text Tables containing lists of genes in the integrated BRCA and GBM KEGG pathways. (PDF)
