# Supplementary material for: Network-Based Integration of Disparate Omic Data To Identify "Silent Players" in Cancer
Source: PLoS Comput Biol. 2015 Dec 18;11(12):e1004595. doi: 10.1371/journal.pcbi.1004595 (PMC4684294; doi:10.1371/journal.pcbi.1004595)
Supplement: S1 Text — (PDF) [file pcbi.1004595.s001.pdf]

Network-Based Integration of Disparate Omic Data To Identify "Silent Players" in Cancer (Supplementary Material)

Matthew Ruffalo[1], Mehmet Koyutürk*[1,2], and Roded Sharan*[3]

[1]*Department of Electrical Engineering and Computer Science, Case Western Reserve University, Cleveland, OH, USA*
[2]*Center for Proteomics and Bioinformatics, Case Western Reserve University, Cleveland, OH, USA*
[3]*School of Computer Science, Tel Aviv University*

*These authors contributed equally to this work.

**Gene**
ABL1
AHR
AKT1
ALKBH1
ANXA1
APOBEC3G
AQP7
AR
ARAF
ATF1
ATM
ATR
AURKA
BACH1
BAD
BAK1
BARD1
BAX
BCL2
BID
BLM
BMPR1A
BMPR2
BRAF
BRCA1
BRCA2
CASP3
CASP8
CASP9
CCNB1IP1
CCND1
CDC25A
CDC25B
CDC42
CDH1
CDK2
CDK4
CDK7
CERK
CHEK1
CHEK2
CHUK
CREB1
CSNK1D
CTNNB1
CYP19A1
DAG1
DCAKD
DHTKD1
E2F1
EDAR
EGFR
EP300
ERAL1
ESR1
FADD
FAU
FER
FILIP1
FOXO1
GADD45A

GDI1
GRIK1-AS2
GRN
GSK3A
HDAC1
HIPK2
HMGCR
IMPA1
IRS1
ITPKC
JAK1
JAKMIP1
JUN
KRAS
LGALS13
LOC100509620
MAP3K13
MAP3K7CL
MAPK1
MAX
MDM2
MMP1
MRE11A
MSH2
MSH6
MTOR
MYC
MYCBP2
MYT1
NAB1
NCOA3
NF1
NFKB1
NOXA1
NUP85
PAK1
PHB
PIAS1
PIGR
PIK3R2
PKIA
PLK1
PLK3
PML
PTEN
RAC1
RAD50
RAD51
RAD54L
RALA
RALGAPA1
RAP1A
RASGEF1A
RASGRP3
RB1
RHEB
RHO
RPP38
RRAS
SELK
SIRT1
SMAD1

SMAD2
SMAD4
SMAD6
SMAD7
SMARCA4
SMEK1
SMEK2
SP1
STAT1
STK11
TAB1
TFPI
TGFBR1
TGFBR2
TP53
TPR
TRADD
TSC1
TSC2
UBE2F
USP15
USP16
USP21
USP38
VEGFA
WEE1
XRCC3
ZMIZ1
ZMYND8
ZNF655

Table A. Genes contained in the integrated BRCA pathway.

**Gene**
AKT1
AKT2
AKT3
ARAF
BRAF
CALM1
CALM2
CALM3
CALML3
CALML5
CALML6
CAMK2A
CAMK2B
CAMK2D
CAMK2G
CCND1
CDK4
CDK6
CDKN1A
CDKN2A
E2F1
E2F2
E2F3
EGF
EGFR
GRB2
HRAS

IGF1
IGF1R
KRAS
MAP2K1
MAP2K2
MAPK1
MAPK3
MDM2
MTOR
NRAS
PDGFA
PDGFB
PDGFRA
PDGFRB
PIK3CA
PIK3CB
PIK3CD
PIK3CG
PIK3R1
PIK3R2
PIK3R3
PIK3R5
PLCG1
PLCG2
PRKCA
PRKCB
PRKCG
PTEN
RAF1
RB1
SHC1
SHC2
SHC3
SHC4
SOS1
SOS2
TGFA
TP53

Table B. Genes contained in the GBM KEGG pathway.
